# Clinical Evaluation of 16-Week Supplementation with 5HMO-Mix in Healthy-Term Human Infants to Determine Tolerability, Safety, and Effect on Growth

**DOI:** 10.3390/nu13082871

**Published:** 2021-08-20

**Authors:** Katja Parschat, Cathrine Melsaether, Kristina Rasch Jäpelt, Stefan Jennewein

**Affiliations:** 1 Chr. Hansen HMO GmbH, 53619 Rheinbreitbach, Germany; 2 Chr. Hansen A/S, 2970 Hoersholm, Denmark; DKCMM@chr-hansen.com (C.M.); DKKBA@chr-hansen.com (K.R.J.); 3 Fraunhofer Institute for Molecular Biology and Applied Ecology (IME), 52074 Aachen, Germany; stefan.jennewein@ime.fraunhofer.de

**Keywords:** 5HMO-Mix, human milk oligosaccharides, clinical study, growth, tolerability, 2′-fucosyllactose, 3-fucosyllactose, lacto-*N*-tetraose, 3′-sialyllactose, 6′-sialyllactose, 2′-FL, 3-FL, LNT, 3′-SL, 6′-SL

## Abstract

Human milk oligosaccharides (HMOs) are complex sugars that occur naturally in human breast milk and provide many beneficial functions. Most formula products lack HMOs or contain only the most abundant HMO, 2′-fucosyllactose; however, benefits of HMOs come from multiple sugars. We therefore developed a mixture of five HMOs (5HMO-Mix) mimicking the natural concentrations of the top five HMOs (5.75 g/L total, comprising 52% 2′-fucosyllactose, 13% 3-fucosyllactose, 26% lacto-*N*-tetraose, 4% 3′-sialyllactose, and 5% 6′-sialyllactose) representing the groups of neutral, neutral-fucosylated, and sialylated HMOs. We conducted the first multicenter, randomized, controlled, parallel-group clinical study assessing the safety, tolerability, and effect on growth of formula containing the 5HMO-Mix in healthy infants. We enrolled 341 subjects aged ≤14 days; 225 were randomized into groups fed either with infant formula containing 5HMO-Mix (5HMO-Mix) or infant formula without HMOs (IF) for 4 months, with the others exclusively breastfed. There were no differences in weight, length, or head circumference gain between the two formula groups. The 5HMO-Mix was well tolerated, with 5HMO-Mix and breastfed infants producing softer stools at a higher stool frequency than the control formula group. Adverse events were equivalent in all groups. We conclude that the 5HMO-Mix at 5.75 g/L in infant formula is safe and well tolerated by healthy term infants during the first months of life.

## 1. Introduction

Breastfeeding is the most effective way to ensure infant health because human breast milk provides a nutritionally complete meal as well as all the bioactive components needed for optimal development during the first months of life [1]. The World Health Organization (WHO) thus recommends exclusive breastfeeding from the first hour after birth to 6 months of age, although this advice is followed for only 44% of infants worldwide and an even lower proportion in industrialized countries because of clinical or social factors [2]. To compensate for the benefits from human breast milk, infant formula products have to be developed containing ingredients that match human breast milk as closely as possible. Human breast milk is mainly composed of water, lactose, lipids, and milk proteins, but human milk is particularly rich in complex carbohydrates, collectively known as human milk oligosaccharides (HMOs), which are nondigestible for humans. Breast milk contains more than 150 structurally distinct HMOs, which can be divided into three classes: neutral fucosylated, neutral nonfucosylated, and acidic or sialylated HMOs [3]. HMOs are known to perform a range of beneficial physiological functions including the establishment and maintenance of a healthy gut microbiome [4,5,6,7,8,9,10,11,12,13,14], the development of the immune system and modulation of inflammatory responses [1,15,16,17], the strengthening of the gastrointestinal barrier [18,19], and the promotion of neuronal development and cognitive function [20,21,22,23,24,25]. These health-promoting effects have been extensively reviewed elsewhere [1,15,26,27]. The beneficial functions of HMOs have been shown to depend on the structure of each specific HMO. As an example, sialylated HMOs and 2′-fucosyllactose (2′-FL) have been reported to impact brain development and learning capacities [22,23,25], nonfucosylated HMOs are a preferred substrate for specific bifidobacteria [8], and HMOs from different classes act as decoys to prevent adhesion of specific viral or bacterial pathogens to receptors on human cells [28]. Navidat et al. [19] recently showed that a blend of six HMOs representing all three classes of HMOs could decrease inflammation-mediated gut barrier disruption by 60%, with 2′-FL being the most effective HMO in the blend. More research and clinical application is necessary to explore defined functions of specific HMOs and to investigate synergistic effects of HMOs in blends.

The concentration and relative abundance of HMOs varies throughout the course of lactation and also varies widely among women due to diverse factors such as geographic location, maternal diet and physiological status, the individual genetics of the mother, gestational age, mode of delivery, and even seasonal influences [29,30,31,32,33,34,35,36]. There are major genetic differences determined by the Secretor (Se) and Lewis (Le) blood group genes, which control the expression of the specific α (1,2), α (1,3), and α (1,4) fucosyltransferases responsible for different fucosylation patterns of glycans. While fucosylated HMOs are the most abundant HMOs in Se+ women (50%; 35% in Se− women), nonfucosylated HMOs represent the most abundant class in Se− women (55%) compared to Se+ (42%). Sialylated HMOs are more equally distributed between Se+ (12%) and Se− women (14%) [37]. Se+ women account for 70–90% of all women [31] and produce large quantities of α (1,2)-fucosylated HMOs such as 2′-FL and lacto-*N*-fucopentaose I. 2′-FL is by far the most abundant HMO found in milk of Se+ women, ranging in concentration from 1.28 to 4.13 g/L over the course of lactation. The most abundant α (1,3)-fucosylated HMO is 3-fucosyllactose (3-FL), ranging in concentrations from 0.06 to more than 2.4 g/L, increasing in concentration postpartum. 3-FL represents the most common neutral fucosylated HMO in Se− breast milk, where 2′FL is lacking. The most abundant sialylated HMOs are 3′-sialyllactose (3′-SL) and 6′-sialyllactose (6′-SL), with concentration ranges of 0.09–0.35 g/L and 0.06–1.3 g/L, respectively. Among the nonfucosylated neutral HMOs, lacto-*N*-tetraose (LNT) is the most abundant, with a concentration range of 0.2–1.7 g/L. These data were compiled by comparing reported HMO concentrations explored in longitudinal studies that analyzed breast milk of mothers that delivered term infants [29,34,36,38,39,40,41]. To provide infant formulae that match the HMO profile of natural breast milk as closely as possible, we developed a mixture of five HMOs (5HMO-Mix) that combines the five most abundant HMOs across all three structural classes, aiming for synergistical and complementary beneficial effects for the infant’s development. The reconstituted 5HMO-Mix comprises 2.99 g/L 2′-FL, 0.75 g/L 3-FL, 1.5 g/L LNT, 0.23 g/L 3′-SL, and 0.28 g/L 6′-SL, reflecting proportions of 52%, 13%, 26%, 4%, and 5%, respectively. As described before, HMO concentrations vary widely among women and during the lactation period. Thus, in this study, we chose concentrations of HMOs that reflected an average of reported physiological HMO concentrations in human breast milk, with a slightly increased proportion of LNT to promote growth of beneficial intestinal bacteria. Thus far, clinical studies have been carried out with 2′-FL or mixtures of two HMOs [42,43,44,45,46], but to the best of our knowledge there have been no clinical studies published on the tolerance and safety of HMO mixtures containing multiple HMOs from all three classes.

We present a multicenter, randomized, controlled, parallel-group clinical study designed to evaluate the safety and tolerability of the 5HMO-Mix and its effect on growth when applied over a 16-week period at a concentration resembling physiological concentrations in human milk. The 5HMO-Mix was well tolerated, resulting in no differences in weight, length, or head circumference gain between the 5HMO-Mix and control formula groups and a stool consistency and frequency more similar to that of breastfed infants.

## 2. Materials and Methods

### 2.1. Clinical Study—General Aspects and Ethical Compliance

This study was a randomized, multicenter, controlled, parallel nutritional noninferiority study performed at 12 sites across Germany, Italy, and Spain between December 2018 and November 2020. Subjects were recruited from two sites in Germany (Neonatologische Intensivmedizin des Klinikum Südstadt, Rostock and Facharztpraxis für Kinder- und Jugendmedizin, Mannheim), five sites in Italy (Ospedale San Raffaele, Milano; ASST Spedali Civili di Brescia–Presidio Ospedale dei Bambini, Brescia; Azienda Ospedaliero Universitaria Consorziale Policlinico di Bari, Bari; Azienda Ospedaliera San Giovanni Addolorata di Roma, Rome; and Instituto Giannina Gaslini di Genova, Genoa), and five sites in Spain (Hospital Joan XXII de Tarragona, Tarragona; Hospital Universitario Sant Joan de Reus, Reus; Hospital HM Puerta del Sur, Madrid; Hospital HM Nuevo Belen, Madrid; and Hospital HM Montepríncipe, Madrid). The study was performed in accordance with the principles of the Declaration of Helsinki and good clinical practice. All procedures involving human subjects were approved by the relevant ethics committees for each site. Written informed consent was obtained from all parents or legal guardians of the subjects before any study-related procedures were initiated. The study was registered on clinicaltrials.gov (ID = NCT03513744) (accessed on 5 June 2021) before enrolling the first subject.

### 2.2. Participants

Eligible subjects were healthy female and male infants ≤14 days of age at visit 1 (V1), born at full term (≥37 and ≤42 weeks of gestational age), singleton birth, with a birth weight of 2500–4500 g and an APGAR score of 9 or 10 as assessed within the first 15 min after birth. Infants were excluded from the study if they had (a) any congenital disorders that could affect the study outcome or subject safety, (b) self-reported parental or sibling allergy to cow’s milk protein, or (c) administration of antibiotics prior to V1 and concurrent treatment with any medicine or natural health products unless medically indicated by a professional healthcare provider. In addition, subjects were excluded if parents were not prepared to feed the subject solely within the assigned study group. The subjects were not allowed to participate in another clinical study that could influence the outcomes of this study prior to V1 or during this study. A complete list of inclusion and exclusion criteria is provided in Table S1.

### 2.3. Study Design

**Figure 1:**
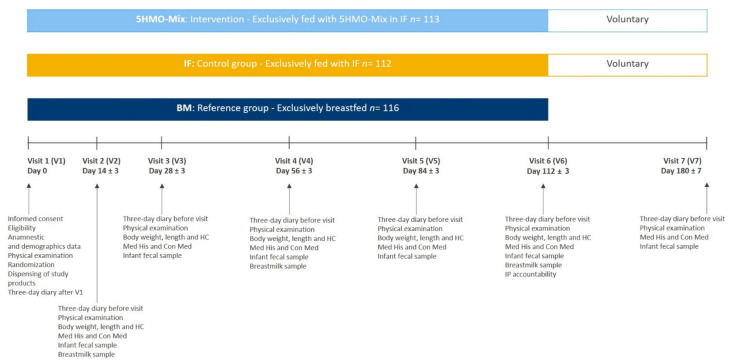
Study flowchart.

The multicenter study comprised three parallel arms consisting of two randomized, double-blinded intervention groups receiving either 5HMO-Mix in infant formula (5HMO-Mix) or infant formula without 5HMO-Mix (IF) and one group of breastfed infants as a reference group (BM). Mothers independently and voluntarily chose to not breastfeed after delivery and before enrollment to the study. The study comprised a 16-week intervention period with six visits (V1–V6) followed by an 8-week voluntary follow-up, and if the parents agreed to continue the follow-up period with the dispensed product, V7 was scheduled at 6 months ± 7 days after V1 (Figure 1). During the intervention and follow-up periods, the parents of the subjects completed a 3-day diary to assess the study outcomes and report compliance with the study treatment.

After obtaining informed consent at V1, we collected demographic (infant and mother) and anamnestic information, conducted a physical examination, and determined baseline anthropometric data (weight, length, head circumference). Infants were allocated to the BM group based on parental/legal guardian choice, and those not allocated to the BM group were randomized to one of the intervention groups (5HMO-Mix or IF). Subsequent visits were scheduled at 14 ± 3 days (V2), 28 ± 3 days (V3), 56 ± 3 days (V4), 84 ± 3 days (V5), and 112 ± 3 days (V6) after V1. Subjects in the BM groups ended the study at V6.

Efficacy was evaluated at all six visits. Safety and tolerability were assessed based on diary entries on the first 3 days after V1 and the 3 days prior to each subsequent visit. All adverse events (AEs), defined as any medical occurrence in a study subject during the intervention period, and concomitant medication and medical treatment or healthcare utilization were also recorded. All data collected during visits were entered by site staff into an electronic case report form (eCRF). Parents were asked to collect stool samples from infants at V1 and in the week prior to each subsequent visit. In the BM group, mothers collecting stool samples were also asked to provide breast milk samples at V2, V4, and V6, sampled either in the week before the visit or on site. The study products were provided to the subjects at each visit, and unused packages had to be returned at the following visit.

Packaging and labeling of study products were carried out by a clinical supplies management company (CSM, Schwalbach, Germany). The randomization list was generated by a statistician at IZKS Mainz (not otherwise involved in the study) using SAS v9.4 (SAS Institute). Randomization to the two intervention groups was performed in a 1:1 ratio in blocks of four and stratified by sex and site. Subject allocation was achieved using a central interactive web-response system RandomiXer hosted by IZKS. All the subjects, parents, investigators, CRO, and sponsor staff involved in the study were blinded until the final database was locked.

### 2.4. Study Product

The 5HMO-Mix (2.99 g/L 2′-FL, 0.75 g/L 3-FL, 1.5 g/L LNT, 0.23 g/L 3′-SL, and 0.28 g/L 6′-SL) was produced by Chr. Hansen HMO GmbH, Rheinbreitbach, Germany. It was mixed with a basic infant formula providing proteins, lipids, and other carbohydrates as well as vitamins and other nutrients. The infant formula was manufactured by Töpfer (Dietmannsried) in Germany under GMP and HACCP procedures, ISF food higher level approved using organic raw materials (besides 5HMO-Mix), and in compliance with Regulation (EU) 2006/141, Regulation (EC) 609/2013, Regulation (EC) 1881/2006, and Regulation (EC) 2073/2005. The final concentration of 5HMO-Mix in the ready-to-use formula was 5.75 g/L and was achieved by partially replacing the carbohydrates (maltodextrin) in the basic formula with the 5HMO-Mix powder (4.35 g/100 g formula powder), resulting in insignificant change of the osmolarity of the formula. The control formula (IF) contained the same quantities of proteins, lipids, vitamins, and other nutrients as the formulated 5HMO-Mix (Table 1, a complete list of ingredients of the formulae is provided in Table S2). The study and control products had a similar appearance, taste, and smell and were provided in identical 300 g bags with identical labeling. Study products were labeled according to the randomization lists and were identified only by the randomization number.

The infants were fed *ad libitum* with only the assigned study product during the entire intervention period. Infants in the reference arm were fully breastfed fed *ad libitum*. Study product intake was calculated from the weight of the delivered and returned packages. A subject was considered compliant with study product intake when they consumed at least 80% of the anticipated quantity as calculated from the average intake by infants aged 0–4 months.

### 2.5. Outcome Measures

The primary outcome was the mean daily body weight increment over a 4-month period, calculated from the subject’s weight at baseline (V1) and at V6 divided by the number of days between the visits. Secondary outcomes included other anthropometric data such as (changes in) weight, length, and head circumference, their increments, and their respective WHO growth standard z-scores (weight-for-age, length-for-age, head circumference-for-age, and weight-for-length). Infant (naked) body weight was measured to the nearest 5 g using a calibrated electronic balance. Infant body length was measured to the nearest 0.1 cm using a length board. Head circumference (the largest occipitofrontal circumference) was measured with a non-stretchable measuring tape. Measurements were carried out twice per visit by the same person. If the difference between the two measurements exceeded 5%, a third measurement was taken, and all three measurements were used for the statistical analysis.

A 3-day paper diary recounting the first 3 days after V1 and the last 3 days prior to visits V2–V6 was used to collect information about tolerability endpoints (defined as stool frequency and consistency), digestive tolerance parameters (regurgitation, vomiting, and flatulence), and behavioral parameters (fussiness, crying, and awakening at night). The total number of stools and stool consistency were assessed using the Amsterdam Stool Form Chart [47]. Regurgitation was rated on a scale with the response options 0 (never), 1 (little, less than a teaspoon), 2 (much, 1–5 teaspoons), and 3 (very much, more than 5 teaspoons). Vomiting was assessed as the number of episodes per day, and flatulence was rated on a scale with the response options 0 (never), 1 (sometimes), and 2 (often). Behavioral parameters (fussiness without crying, crying, and awakening at night) were rated on a scale with the response options 0 (never), 1 (sometimes), and 2 (often). Compliance and estimated intake of the 5HMO-Mix and IF were assessed by parents recording the total daily intake in the diary. Diaries were reviewed for completeness by the study personnel at each visit.

AEs and concomitant medication and healthcare utilization were assessed at each visit and documented in the eCRF. AEs were coded and grouped according to the Medical Dictionary for Regulatory Activities (MedDRA) by primary system, organ, and class (SOC) and preferred terms (PT) and categorized by seriousness, intensity, and causal relationship to the study products. Concomitant medication was encoded and classified by interposed verbatim terms using the WHO anatomical therapeutical chemical (ATC)/defined daily dose (DDD) index.

### 2.6. Statistical Analysis

All statistical analyses were carried out using SAS v9.4. The safety dataset (SS) consisted of all subjects who were randomized to one of the two intervention groups or enrolled in the breastfed reference group and received at least one feeding (formula or breast) for which there was any tolerability data available up to V6. The full-analysis dataset (FAS) included all subjects of the safety dataset for whom there was at least one value of the body weight at baseline and after baseline available. The per-protocol dataset (PPS) included all subjects from the FAS without any major deviations. Major deviations included noncompliance with the assigned group for ≥3 consecutive days, complementary foods intake above ≥4 teaspoons of complementary foods in total per day for ≥3 consecutive days, use of a calorie-containing product for medical reasons for ≥3 consecutive days, no data on primary endpoint (body weight) available after baseline, and no availability of diary data. The main goal of this study was to demonstrate the ability of IF containing five different HMOs in their natural concentrations and ratios to support normal growth within the first 4 months of life by showing its noninferiority compared to the standard formula in terms of body weight increment from baseline to month 4. The 5HMO-Mix was considered noninferior to IF given a body weight gain in the 5HMO-Mix group of more than -3 g/day compared to the IF group [48]. In other words, noninferiority was achieved when the lower limit of the one-sided 97.5% confidence interval (CI) for the body weight increment at month 4 (5HMO-Mix vs. IF) was greater than -3 g/day. Assuming an expected difference in means of 0 g/day and a common standard deviation (SD) in body weight gain of ~6 g/day for male and female infants [49,50], a sample size of 64 subjects per group was needed for a two-group *t*-test with a 2.5% one-side significance level to achieve 80% power as calculated using nQuery Advisor v7.0 (Statsols). Considering a 40% dropout rate, the enrollment of 108 subjects per study arm was planned to reach the number of 64 subjects in the PPS. For the confirmatory analysis of the primary outcome, we used a statistical analysis of covariance (ANCOVA) based on independent, normally-distributed random variables with equal variances. The dependent variables were treatment group and stratification factor (sex and site) as fixed effects and body weight at baseline as a covariate. The primary endpoint was analyzed for the FAS and PPS. In addition to the absolute data on body weight, length, and head circumference, growth parameters were complemented by the evaluation of age-specific and sex-specific WHO child growth standard z-scores that were calculated using the WHO Anthro Survey Analyzer (2011). Secondary and exploratory outcomes were analyzed using an ANCOVA model similar to that used for the primary outcome variable at each visit of the FAS and PPS using original data or data after multiple imputation (using the SAS procedures MI and MIANALYZE) if data points were missing. The number of imputations was set to 100, and the covariates considered for inclusion were the treatment group, site, sex, birth weight, age, and weight at all time points.

Due to the COVID-19 pandemic in spring/summer 2020, the assessments were carried out in accordance with the relevant guidelines [51,52]. On-site visits and the on-site assessment of anthropometric data were replaced with telephone consultations, and the assessment of body weight, length, and head circumference were conducted by parents or pediatricians. During each telephone consultation, a questionnaire was completed that prompted for data and information about how measurements were taken. To evaluate the quality of these off-site measurements, sensitivity analysis was applied to the primary endpoint body weight, treating the off-site data as missing data and performing a multiple imputation as described above.

## 3. Results

### 3.1. Subject Characteristics

We screened 341 term infants at two centers in Germany, five centers in Italy, and five centers in Spain. All 341 infants were enrolled. We allocated 116 infants (34.0%) to the BM group based on requests from the parents or legal guardians and 225 (66.0%) to the formula groups (5HMO-Mix or IF). Among the latter, 113 infants (33.1%) infants were randomized to the 5HMO-Mix group and 112 (32.8%) to the IF group.

**Figure 2:**
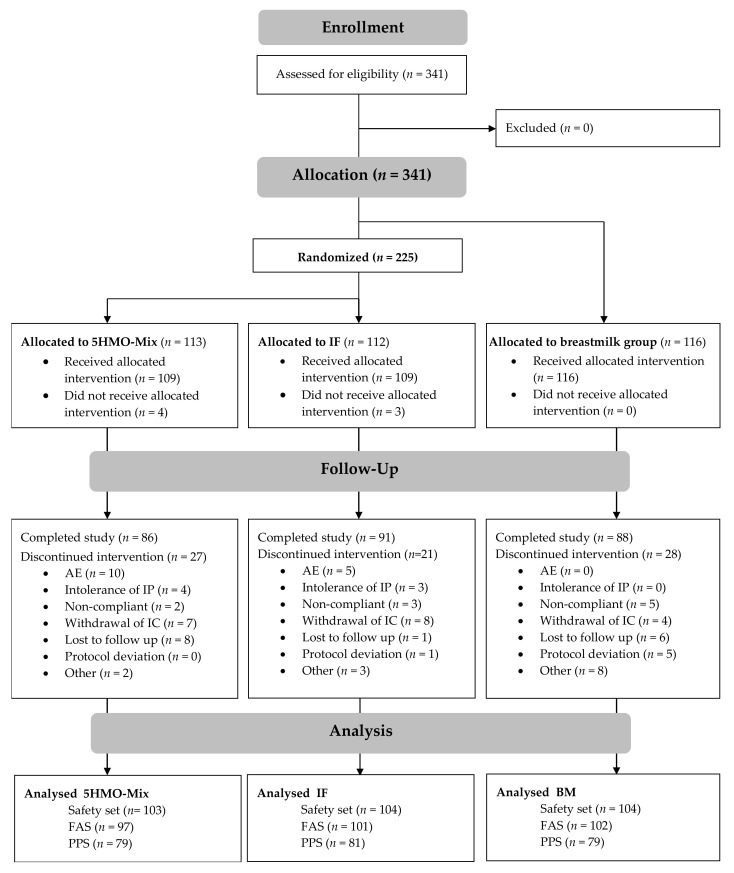
Consort diagram showing the disposition of subjects.

The number of infants that completed the study was 265 (77.7%): 86 (76.1%) in the 5HMO-Mix group, 91 (81.3%) in the IF group, and 88 (75.9%) in the BM group (Figure 2). All subjects randomized to one of the two formula arms or enrolled in the BM group, and who received at least one feed for which tolerability data were recorded up to V6, were included in the safety data set (SS). Furthermore, 300 infants (88.0%) were included in the FAS and 239 (70.1%) in the PPS. The primary endpoint was reported in both the FAS and PPS because both datasets are equally important in a noninferiority trial. Growth parameters were presented for the FAS and tolerability parameters for the SS.

There were no significant differences among the three groups with respect to baseline demographics (Table 2).

### 3.2. Growth Outcomes

The primary endpoint (mean daily body weight gain after 4 months intervention) was assessed in the FAS and PPS. In the 5HMO-Mix group, the mean body weight increase from V1 to V6 was 3347.7 ± 667.0 g (FAS) and 3329.8 ± 670.9 (PPS) with a daily mean body weight increment of 29.9 ± 5.8 g/day (FAS) and 29.8 ± 6.0 g/day (PPS). The two-sided 95% CI ranged from −0.7 to 2.4 g/day (FAS) and from −0.8 to 2.3 g/day (PPS), with the lower bound above the noninferiority margin of −3 g/day in both populations. The primary endpoint noninferiority of the 5HMO-Mix was therefore observed in the FAS (*p* < 0.001) and the PPS (*p* < 0.001) versus IF, with respect to mean daily body weight gain. Average body weight in the two intervention groups from V1 to V6 is shown in Table 3.

There were no significant differences in mean weight, length, or head circumferences between the two intervention groups during the study (data for length and head circumference not shown). At V6 we observed differences between the 5HMO-Mix and BM groups (6578.8 vs. 6391.0 g, *p* = 0.0496) and between the IF and BM groups (6557.3 vs. 6391.0 g, *p* = 0.0431) with respect to body weight, but there were no significant differences in body weight between the intervention groups and BM group at other time points. At both V5 and V6, we observed significant differences between the 5HMO-Mix and BM groups (*p* = 0.014 and *p* = 0.0019, respectively) and the IF and BM groups (*p* = 0.0383 and *p* = 0.0006, respectively) with respect to body length in the FAS population, but there were no significant differences when comparing the 5HMO-Mix and IF groups at all time points. We observed no significant differences in the change of head circumference measures among any of the groups at any time point.

**Figure 3:**
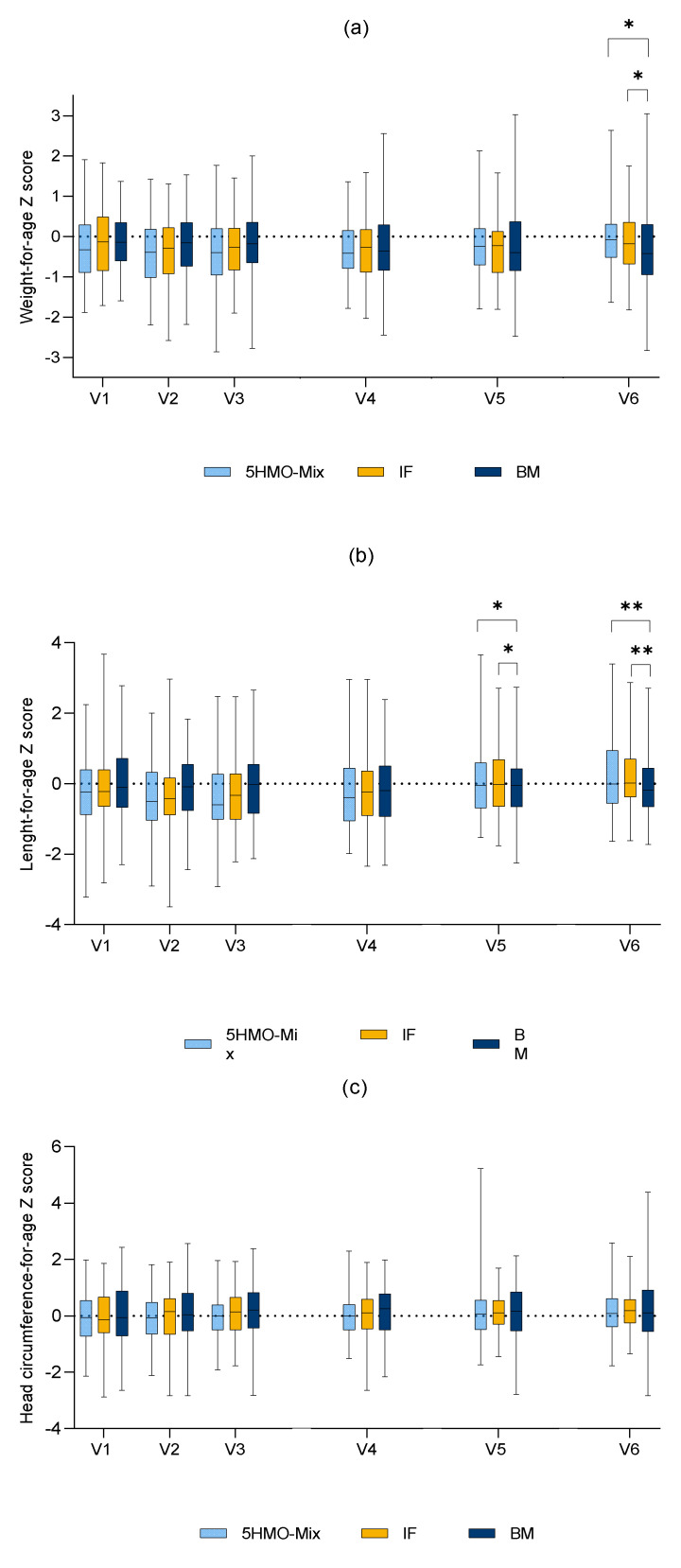
WHO growth standard z-scores weight-for-age (**a**), length-for-age (**b**), and head circumference-for-age (**c**) per visit. Full analysis set. Explorative *p*-values: * *p* < 0.05, ** *p* < 0.01. Abbreviations: 5HMO-Mix = 5HMO-Mix in infant formula, IF = infant formula without 5HMO-Mix, BM = breastfed infants.

No significant differences in weight-for-age z-scores were observed between the 5HMO-Mix and IF groups at any time point between V2 and V6 in the FAS. Compared to the BM group, the mean z-scores were slightly higher in both intervention groups at V6. For the remaining time points, there were no significant differences between either of the intervention groups and the BM group. The length-for-age z-score in the FAS differed between the IF and BM groups at V2 (the value was slightly higher in the IF group), but no significant differences were observed in the PPS. No significant differences in length-for-age scores were observed between the two intervention groups in the FAS at any of the time points between V2 and V6. Compared to the BM group, the mean z-scores were slightly higher in the intervention groups at both V5 and V6. There were no significant differences in the head circumference-for-age scores among the study groups in the FAS. The infants fed with the 5HMO-Mix maintained their weight-for-age z-score. The age-based weight, length, and head circumference z-scores according to WHO standard growth curves are shown in Figure 3.

### 3.3. Infant Formula Intake

The mean daily volume intake of 5HMO-Mix and IF were similar in the two intervention groups. Compliance was calculated based on the weight of the returned packages of infant formula. An infant was considered compliant when at least 80% of the expected amount of infant formula was used. Only three infants (3.1%) in the 5HMO-Mix group and four (4.0%) in the IF group were considered noncompliant. The mean daily intake of infant formula (mL) including mean daily kcal intake at the different time points is shown in Table 4. The consumption and estimated energy intake appeared to be comparable.

Average intake of the 5HMO-Mix is presented in Figure S1 in the Supplementary Materials. The mean HMO intake per day was calculated to 2.64 ± 0.79 g (V1), 3.46 ± 0.77 g (V2), 4.28 ± 0.94 g (V3), 4.62 ± 0.87 g (V4), 4.94 ± 1.09 g (V5), and 5.19 ± 0.97 g (V6).

### 3.4. Gastrointestinal and Behavioral Tolerance Parameters

Stool frequency of infants in the 5HMO-Mix and IF groups was an average of 3.9 ± 1.4 and 3.4 ± 1.6 stools per day, respectively, during the first week of intervention, which declined throughout the study to 1.8 ± 1.0 and 1.6 ± 0.9 stools per day, respectively. Changes in stool frequency followed the same pattern in both groups (Figure S2). The infants in the BM group passed more stools per day during the first 28 days of intervention (*p* = 0.0000 at V2 and V3), thereafter following the same decline as observed in the 5HMO-Mix and IF groups. No significant differences between 5HMO-Mix and BM groups were observed from V4 to the end of the intervention. The IF group passed fewer stools per day than the 5HMO-Mix and BM groups at V6 (*p* = 0.0428 and *p* = 0.0136, respectively).

We observed no differences in stool consistency between the two formula groups, but the breastfed infants passed more watery stools more frequently than both other groups. Soft stools were observed significantly more frequently in the 5HMO-Mix group compared to the IF group at V1–V4 (*p* = 0.023, *p* = 0.002, *p* = 0.004, and *p* = 0.045, respectively). However, the number of soft stools in the BM group exceeded those of both intervention groups at nearly all time points (Figure S3). The mean number of formed stools per subject at each visit was similar in all three groups throughout the intervention (Figure S3). The presence of blood stains in stool (hematochezia) was assessed from both the diary and the AE records. Blood stains were observed in seven infants (6.8%) from the 5HMO-Mix group, two (1.9%) from the IF group, and two (1.9%) from the BM group. For the gastrointestinal tolerance parameters, we observed no differences among the three groups in the frequency of flatulence. The mean total score for regurgitation was slightly higher in the 5HMO-Mix group compared to the IF group at all visits except V2 and compared to the BM group at V1. At all other time points, the 5HMO-Mix and BM groups appeared comparable (Figure S4). Vomiting was similar in both formula groups at all time points but was less frequent in the BM group at V1, V2, and V3 (Figure S4).

No difference in fussiness without crying was observed among the three groups during the 4 months of intervention, but a slightly lower incidence at baseline was observed for the 5HMO-Mix group compared to the BM group (*p* = 0.0369). Crying was less frequent in the 5HMO-Mix group compared to the IF group at V1 (*p* = 0.0350) but there were no significant differences between these groups at other time points. Crying was also less frequent in the 5HMO-Mix group compared to the BM group at V4 and V6 (*p* = 0.0132 and *p* = 0.0140, respectively), but there were no differences between the IF and BM groups. Infants in the formula groups woke less frequently at night compared to breastfed infants throughout the investigation period (Figure S5).

### 3.5. Adverse Events

The calculated two-sided 95% CIs for the total incidence of AEs and serious adverse events (SAEs) were comparable in all three study groups. The total incidence of AEs was similar in the 5HMO-Mix and IF groups, both being slightly higher compared to the BM group. The total incidence of SAEs was similar in the 5HMO-Mix and BM groups, both being lower than in the IF group. The total number of reported AEs was 815, among which 50 were deemed related to the study product (relationship = possible, probable, definite, or not assessable/missing), and these were equally distributed in the 5HMO-Mix and IF groups. The incidence of AEs was similar in the 5HMO-Mix and IF groups: 335 AEs in 83 (80.6%) of the 5HMO-Mix infants and 289 AEs in 84 (80.8%) of the IF infants. In the BM group, 191 AEs were recorded in 73 (70.2%) of the infants. Among the 815 AEs, 168 were deemed mild, 64 moderate, and 8 severe. The number and intensity of the reported events were similar among the three groups.

A slightly higher incidence of atopic dermatitis was reported in the IF group (*n* = 7) compared to the 5HMO-Mix group (*n* = 0; *p* = 0.0141), whereas more genital fungal infections were reported in the 5HMO-Mix group (*n* = 5) compared to the IF group (*n* = 0; *p* = 0.0290). Hematochezia was more frequent in the 5HMO-Mix group compared to the BM group, vomiting occurred more frequently in both intervention groups than the BM group, and plagiocephaly occurred more frequently in the 5HMO-Mix group than the BM group. Overall, no significant differences for specific AEs were observed between the two intervention groups or when comparing either intervention group to the BM group. The overall incidence and intensity of AEs potentially linked to the use of formula is summarized in Table 5. For all AEs assessed during the study, please see Table S3.

Sixteen SAEs were reported during the study, four of which (in four infants) were deemed related to the investigational product. Three SAEs were reported in the 5HMO-Mix group, representing 2.9% of the subjects, compared to nine (7.7%) in the IF group and four (3.8%) in the BM group. Two SAEs each in the 5HMO-Mix and IF groups were deemed related to the investigational product. One subject receiving the 5HMO-Mix was hospitalized due to choking and gastroesophageal reflux but recovered. The formula was not withdrawn, and the subject continued in the study. The second subject in the 5HMO-Mix group experienced severe diarrhea; the formula was withdrawn, and the infant was treated with hydrolyzed milk before leaving the study. Both SAEs in the IF group resulted in diagnosis with allergy to bovine milk protein. The subjects recovered after treatment with saline and hydrolyzed milk but left the study prematurely.

Concomitant healthcare utilization was less frequent in the 5HMO-Mix group than the IF group (*p* = 0.0117), but we observed no differences when comparing the intervention groups to the BM group. Healthcare utilization following an AE was comparable between the 5HMO-Mix and IF groups, and between the 5HMO-Mix and BM groups, but less healthcare utilization was observed in the BM group compared to the IF group (*p* = 0.0254).

## 4. Discussion

Although breast milk provides the complete nutritional demands of a growing human infant, the majority (56% globally) of infants are fed on formula products because of a range of clinical and/or social factors that prevent or discourage breastfeeding. Until recently, such formula products provided nutritional sustenance but not the additional beneficial functions attributed to HMOs, as the oligosaccharides in the milk of domestic dairy animals such as cows, goats, and sheep are neither as diverse nor as complex as those found in human breast milk [53]. Over the last two decades, efforts have been made to synthesize HMOs and add them to formula products, but only a limited number of HMOs not representing all structural classes have been tested in the clinic. These earlier studies investigated the safety, tolerability, and effect on growth of 2′-FL [42,43,44], 2′-FL plus LNnT [45,54], or 2′-FL plus 3′-galactosyllactose (3′-GL) [46], typically combined with other prebiotics [42,43,46] such as galacto-oligosaccharides (GOS) and fructo-oligosaccharides (FOS) or probiotics [46,54].

Given that the different natural benefits of HMOs are linked not to a single molecule but rather to a collection of oligosaccharides with diverse chemical properties, we evaluated the safety, tolerability, and effect on growth of a 5HMO-Mix. The mix provides the five most abundant HMOs across all three major chemical categories and in proportions that resemble their natural concentrations and ratios (5.75 g/L in total), without any other prebiotic or probiotic ingredients. The main goal of the study was to investigate the ability of the 5HMO-Mix as part of a basic infant formula to support normal growth in healthy term infants (evaluated in terms of weight gain) compared to a standard formula lacking HMOs. The trial was designed as a noninferiority comparison, defined as at least equivalent performance between the 5HMO-Mix and IF groups.

The primary outcome was the mean daily body weight increment over a 4-month period, calculated from the subject’s weight at baseline and final visit. We observed no significant difference in weight gain between the 5HMO-Mix and IF groups, indicating that the 5HMO-Mix was noninferior to the IF lacking HMOs. The observed mean values for daily weight gain of ~28.7 g/day were similar to those reported in studies comparing IF with 2′-FL plus GOS [42], 2′-FL plus LNnT [45], or 2′-FL plus 3′-GL and GOS/FOS [46]. When comparing the daily weight gain in the two formula groups to the BM group, we observed a slightly higher increase from baseline to V6 for 5HMO-Mix vs. BM (2.1 ± 0.9 g/day, *p* = 0.0199) compared to IF vs. BM (1.6 ± 0.9 g/day, *p* = 0.0674) in the FAS (*n* = 300), but this effect was not seen in the PPS (*n* = 239). Faster weight gain has previously been reported for infants bottle-fed with formula [46,55,56,57]. However, this may be associated with loss of the infant’s self-regulation of energy intake due to early bottle feeding and is also observed when human breast milk is fed by bottle [56,57,58]. Equivalent weight gain was observed in infants fed with formula containing 2′-FL with a lower caloric intake and breastfed infants [42]. The infant formula containing the 5HMO-Mix and the infant formula without 5HMO-Mix used in this study had energy values of ~68 and ~69 kcal/dL, respectively, and therefore a slightly higher mean energy value than human breast milk [59,60]. The slightly higher weight-to-age z-scores for the formula-fed subjects are therefore in line with observations that bottle-fed infants gain weight more quickly.

The HMO intake ranged from 2.6 ± 0.79 g/day at V1 to 5.2 ± 0.7 g/day at V6, showing only a slight increase after 4 weeks. The total HMO concentration and the ratio of the HMOs in the formula remained constant during the intervention period, whereas in breast milk the concentration of several HMOs decreases within the first few months after delivery [40], while a few HMOs, such as 3-FL, become more abundant [29,40]. The absolute concentration of different HMOs is primarily influenced by the mother’s Secretor status but also by her BMI and diet as well as several other factors described above [40,61]. The overall concentration of HMOs declines from 20–25 g/L in the colostrum to 10–15 g/L in mature milk ([33,34] and references therein). Thus, supplying a formula with constant HMO concentration and ratio of different HMOs could result in lower intake of specific HMOs for very young infants and higher amounts of HMOs consumed in the later stage of the study compared to breastfed infants. More personalized HMO compositions for infants at different ages could bring formula products even closer to breast milk. However, more research is needed to gain insight into the structure–function relationship of HMOs and their specific effects in different stages on the infant’s development. The question of whether the mother’s specific HMO profile is beneficial to the offspring is also still to be answered. Breast milk samples from breastfeeding mothers in this study will be analyzed to determine their HMO content and composition in order to find correlations between HMO profiles and the developing infant gut microbiome in mother–child dataset pairs.

The safety of the 5HMO-Mix was confirmed by the similar frequency of AEs in the two formula groups, which was slightly but not significantly higher than that for the BM group. Our study did not intend to investigate specific health outcomes, so parents reported on specific observations only for 3 days after V1 and 3 days before V2–V6, and AEs were recorded at the visits. Earlier studies of formulae containing HMOs revealed a lower incidence of respiratory tract infections and bronchitis, as well as the less frequent use of antibiotics and antipyrenics [45,62]. We did not observe a lower incidence of infections in our 5HMO-Mix group. We did observe slightly higher incidences of gastrointestinal disorders and skin and subcutaneous tissue disorders in the two groups receiving formula products. However, AEs causally linked to the formula products occurred at a similar frequency in both groups. Gastrointestinal disorders are generally the most common AEs in studies of infant formulae [46,63]. However, caregivers in the 5HMO-Mix group were less likely to need healthcare interventions during the study than those in the IF group. SAEs causally linked to the IF products were diagnosed as allergy to bovine milk protein. Studies investigating the use of 2′-FL in a formula with partly or fully hydrolyzed milk protein have already demonstrated that those formulae are safe and well tolerated [44,46,64]. Although hematochezia was more frequently reported in the 5HMO-Mix group, the overall frequency was still very low, and there were no specific safety concern indicators. The hematochezia could be explained by anal fissures, transient firm stool consistency, dyspepsia, gastroenteritis due to contact with pathogenic bacteria or viruses, or allergy to bovine milk protein. We cannot therefore assume a relationship between the frequency of hematochezia and HMO intake.

The mean total number of stools in the 5HMO-Mix group was somewhat higher than that in the IF group for all study visits except V4 but was lower than that in the BM group at V2 and V3. The mean number in the BM group was higher than that in the IF group at all visits. The mean total number of watery stools was comparable in both formula groups at all time points but was higher in the BM group. Infants in the 5HMO-Mix group were more likely to produce soft stools from V1 to V4 compared to infants in the IF group, but the breastfed infants produced softer stools than infants in the formula groups at all time points. The mean total number of firm and hard stools was comparable in all three groups at all time points, although the number of subjects with this stool type was lower in the BM group. The more frequent and generally softer stools in the 5HMO-Mix vs. IF group may reflect the greater similarity between the 5HMO-Mix formula and breast milk, replicating the prebiotic effect. To investigate the effect of the HMOs on the development of the gut microbiome, stool samples were collected from all three groups and will be subject to deep sequencing to identify the dominant microbial communities.

We observed no differences among the three groups in the incidence of regurgitation or flatulence, but the breastfed infants showed a lower frequency of vomiting compared to the other groups at V1–V3. In terms of behavioral parameters, we observed no differences among the three groups for the parameter of fussiness without crying, but infants in the 5HMO-Mix group cried less than breastfed infants, and both groups of bottle-fed infants woke less frequently at night compared to breastfed infants (with no significant difference between the two types of formula). Bottle-fed infants have previously been shown to sleep for longer periods at night than breastfed infants [63].

The study enrolled more than 300 subjects and therefore had sufficient strength to test the 5HMO-Mix formula against a standard comparator with breastfed infants as a reference. However, the study also had several limitations. Tolerability parameters were documented for only 3 days after V1 and 3 days before each subsequent visit. The digestive and behavioral parameters could have been recorded more frequently to provide a clearer outcome. Because of the COVID-19 pandemic in spring/summer 2020, several subjects (particularly those in Spain) were unable to attend on-site visits, and an emergency plan was developed to replace these with telephone visits in accordance with guidelines issued by the FDA and EMA. However, the responsibility for measuring anthropometric data was transferred from professional personnel to the parents or pediatricians, not always precisely in line with the prescribed visit times. We therefore treated the data as missing, and sensitivity analysis was applied by multiple imputations. However, when comparing the original and imputed data, we did not detect significant differences (data not shown).

By implementing the five most abundant HMOs in natural concentrations in an infant formula, we took the next step toward bringing formula products closer to breast milk in terms of carbohydrate diversity. Further studies are needed to investigate health-related outcomes when using this blend of HMOs (5HMO-Mix); using fecal samples collected in this study, we will investigate the effect on modulation of the microbiome. Future studies should determine if even higher numbers of HMOs or specific symbiotic combinations with HMOs have even more significant benefits on the infant’s development. In addition, a multiplicity of in vitro studies support the knowledge on potential health benefits of single HMOs in different areas such as microbiome development, immune modulation, or intestinal barrier function.

## 5. Conclusions

We conclude that an infant formula fortified with a mixture of the five most abundant HMOs (2′-FL, 3-FL, LNT, 3′-SL, and 6′-SL) at the concentrations and ratios resembling those in breast milk supports normal infant growth and is safe and well tolerated for use in healthy term infants.

## Figures and Tables

**Table 1:** Energy, macronutrients, and HMOs in 5HMO-Mix formula and the control IF (per 100 g formula powder and per 100 mL ready-to-drink). Nutritional values for micronutrients are provided in Table S2 in the Supplementary Materials.

		5HMO-Mix		IF	
	Unit	Per 100 g Powder	Per 100 mL Ready to Drink	Per 100 g Powder	Per 100 mL Ready to Drink
Energy ^1,2^	KJ	2129	280	2163	283
	kcal	507	67	515	68
Fat	g	27	3.6	27	3.6
Saturated	g	9.3	1.2	9.3	1.2
Monounsaturated	g	12.6	1.7	12.6	1.7
Polyunsaturated	g	5.1	0.7	5.1	0.7
Carbohydrate ^3^	g	53	7.2	57	7.2
Lactose	g	39	5.2	39	5.2
Maltodextrin	g	11.65	4.8	16	5.4
HMO	g	4.35	0.575	-	-
2′FL	g	2.26	0.299	-	-
3-FL	g	0.57	0.075	-	-
LNT	g	1.13	0.150	-	-
3′-SL	g	0.17	0.023	-	-
6′-SL	g	0.22	0.028	-	-
Protein	g	11	1.4	11	1.4
Salt ^4^	g	0.45	0.060	0.45	0.06

^1^ These variations were within natural and tolerable ranges and met the requirements of Regulation (EU) 1169/2011 regarding the tolerances for nutritional values indicated on the label. ^2^ The energy of the blinded formulae was declared as 513 kcal/100 g formula powder. ^3^ The amount of carbohydrates in the blinded formulae was declared as 55 g/100 g formula powder. ^4^ Salt content exclusively due to the presence of naturally occurring sodium in the raw materials and minerals. HMO: Human milk oligosaccharides; 2′-FL: 2′-fucosyllactose; 3-FL: 3-fucosyllactose; LNT: lacto-*N*-tetraose; 3′-SL: 3′-sialyllactose; 6′-SL: 6′-sialyllactose

**Table 2:** Demographic and clinical characteristics of infants (safety set, *n* = 311).

	5HMO-Mix *n* = 103	IF *n* = 104	BM *n* = 104	Total *n* = 311
Sex, *n* (%)				
Male	54 (52.4)	55 (52.9)	52 (50.0)	161 (51.8)
Female	49 (47.6)	49 (47.1)	52 (50.0)	150 (48.2)
Age at enrollment, days	4.8 ± 3.2 (1–16)	4.3 ± 2.7 (1–14)	5.2 ± 3.6 (1–14)	4.8 ± 3.2 (1–16)
Gestational age at birth, weeks	39.3 ± 1.1	39.3 ± 1.2	39.4 ± 1.1	39.3 ± 1.1
Ethnicity, *n* (%)				
Caucasian	95 (92.2)	100 (96.2)	97 (93.3)	292 (93.9)
African	1 (1.0)	1 (1.0)	-	2 (0.6)
Mixed	6 (5.8)	3 (2.9)	4 (3.8)	13 (4.2)
Other	1 (1.0)	-	3 (2.9)	4 (1.3)
Mode of delivery, *n* (%)				
Vaginal	56 (54.1)	64 (61.5)	73 (70.2)	193 62.1)
Cesarean section	32 (31.1)	33 (31.7)	24 (23.1)	
Assisted vaginal	15 (14.6)	7 (6.7)	7 (6.7)	29 (9.3)
Birth weight, g	3321.8 ± 434.7	3351.1 ± 405.5	3412.0 ± 381.3	3361.8 ± 408.1
Birth length, cm	50.04 ± 2.01	50.44 ± 2.23	50.54 ± 1.97	50.34 ± 20.8
Birth head circumference, cm	34.65 ± 1.13	34.68 ± 1.23	34.65 ± 1.48	34.66 ± 1.29
APGAR score ^1^	10.0 ± 0.2 (9–10)	9.9 ± 0.3 (9–10)	9.9 ± 0.3 (9–10)	9.9 ± 0.3 (9–10)
Use of antibiotics by mother before or during delivery, *n* (%)	15 (14.6)	20 (19.2)	22 (21.2)	
Feeding history from birth up to enrollment, *n* (%)				
Breast milk fed	11 (10.7)	6 (5.8)	93 (92.3)	
Infant formula fed	92 (89.3)	98 (94.2)	8 (7.7)	

^1^ Mean ± SD (min–max)

**Table 3:** Average body weight (g) at V1, V2, V3, V4, V5, and V6 for the two intervention groups. Data shown for subjects with values at V1 and at the actual visit were included in the analysis. ANCOVA with the fixed effect factors “treatment”, “site”, and “sex” and body weight at V1 as covariate was used for the statistical analysis (observed cases, FAS).

	5HMO-Mix				IF				
	*n*	Mean ± SD	Min	Max	*n*	Mean ± SD	Min	Max	*p*-Value
V1	97	3236.5 ± 410.5	2480.0	4260.0	101	3274.4 ± 393.9	2510.0	4230.0	-
V2	97	3643.2 ± 433.0	2863.3	4690.0	100	3682.5 ± 444.1	2686.7	4790.0	0.8441
V3	91	4212.0 ± 477.5	3271.7	5710.0	97	4257.1 ± 468.8	3306.7	5316.7	0.9579
V4	88	5148.7 ± 537.1	3995.0	6540.0	93	5178.9 ± 542.8	3906.7	6280.0	0.8589
V5	86	5893.7 ± 623.9	4595.0	8000.0	88	5867.0 ± 600.4	4603.3	7490.0	0.4245
V6	84	6578.8 ± 697.6	5170.0	9195.0	89	6557.3 ± 672.8	5059.3	8280.0	0.5314

**Table 4:** Average daily intake of infant formula (mL/day) and average kcal intake after V1 and before V2, V3, V4, V5, and V6 (FAS).

	5HMO-Mix					IF				
	*n*	Mean ± SD	Median	Min	Max	*n*	Mean ± SD	Median	Min	Max
V1	93					101				
mL		459.7 ± 137.7	455.0	23.3	990.0		442.9 ± 135.4	440.0	150.0	811.7
Kcal		312.6 ± 93.6	309.4	15.9	673.2		301.2 ± 92.1	299.2	102.0	551.9
V2	95					100				
mL		603.5 ± 134.5	605.0	70.0	900.0		617.3 ± 141.6	604.2	80.0	1196.7
Kcal		410.4 ± 91.5	411.4	47.6	612.0		419.7 ± 96.3	410.8	54.4	813.7
V3	89					97				
mL		744.5 ± 163.2	713.3	453.3	1456.7		769.7 ± 161.6	766.7	410.0	1440.0
Kcal		506.3 ± 111.0	485.1	308.3	990.5		523.4 ± 109.9	521.3	278.8	979.2
V4	87					94				
mL		802.4 ± 152.1	786.7	490.0	1290.0		832.7 ± 179.9	808.3	533.3	1910.0
Kcal		545.6 ± 103.4	534.9	333.2	877.2		566.2 ± 122.3	549.7	362.7	1298.8
V5	86					92				
mL		857.6 ± 189.4‚	835.8	466.7	1430.0		871.2 ± 185.4	855.0	556.7	1591.7
Kcal		583.2 ± 128.8	568.4	317.3	972.4‚		592.4 ± 126.0	581.4	378.5	1082.3
V6	83					89				
mL		902.5 ± 170.0	870.0	480.0	1580.0		928.2 ± 165.9	900.0	636.7	1576.7
Kcal		613.7 ± 115.6	591.6	326.4‚	1074.4		631.2 ± 112.8	612.0	432.9	1072.1

**Table 5:** Number of infants in the safety set (*n* = 311) with the most abundant adverse events reported during the study period (V1–V6) categorized by MedDRA v23.1. Primary SOC. Please see Table S3 for full overview of all AEs.

	5HMO-Mix	IF	BM	5HMO-Mix vs. IF	5HMO-Mix vs. BM	IF vs. BM
	*n*	*n*	*n*	*p*-Value	*p*-Value	*p*-Value
Gastrointestinal disorders	64	58	40	0.3975	0.0008	0.0180
Infections and infestations	32	28	34	0.5426	0.8817	0.4486
Skin and subcutaneous tissue disorders	14	21	7	0.2660	0.1129	0.0074
Respiratory, thoracic, and mediastinal disorders	10	10	7	1.0000	0.4603	0.6140
General disorders and administration site conditions	6	8	15	0.7831	0.0635	0.1838
Metabolism and nutrition disorders	5	1	3	0.1187	0.4981	0.6214

*n* = number of subjects.

## Supplementary Materials

The following tables and figures are available online at https://www.mdpi.com/article/10.3390/nu13082871/s1, Table S1: Inclusion and exclusion criteria, Table S2: Energy, nutrients, and HMOs in 5HMO-Mix formula and the control IF, Table S3: Complete overview of number of infants in the safety set with adverse events during the study period, Figure S1: Average intake of the 5HMO-Mix at different timepoints, Figure S2: Average stool frequency per day, Figure S3: Stool consistency, Figure S4: Score values for reguration, vomiting, and flatulence. Figure S5: Score values for behavioral parameters.
