# Supplementary material for: Clinical Evaluation of 16-Week Supplementation with 5HMO-Mix in Healthy-Term Human Infants to Determine Tolerability, Safety, and Effect on Growth
Source: Nutrients. 2021 Aug 20;13(8):2871. doi: 10.3390/nu13082871 (PMC8401119; doi:10.3390/nu13082871)
Supplement: Supplementary file 1 [file nutrients-13-02871-s001.zip › nutrients-1294055-supplementary.pdf]

Supplementary

Table S1. Inclusion and exclusion criteria.

Inclusion Criteria

1. Healthy male or female
2. Singleton birth
3. Full-term infant (≥ 37 and < 42 weeks of gestational age)
4. APGAR (Appearance, Pulse, Grimace, Activity and Respiration) score of 9 or 10, at the latest assessment within first 15 min after birth
5. Birth weight 2500–4500 g
6. 1–13 days old at V1 (1 = day of birth)
7. BM group only: parent(s')/legal representative(s') readiness to solely feed the subject with mother's breast milk during the study
8. Formula groups only: parent(s')/legal representative(s') readiness to solely feed the subject using the assigned IP during the study (at least until V6), following the unequivocal decision for the mother prior to the study not to breast-feed the infant
9. Parent(s')/legal representative(s') readiness not to administer vitamin or mineral supplements (with the exception of those recommended by a medical care provider), solid foods or any other source of calories to the subject during the study period from V1 to V6
10. Parent(s')/legal representative(s') readiness and ability to:
    a. fill in the diary
    b. attend scheduled visits
    c. comply with the protocol requirements
11. Participation was based upon written informed consent by the parent(s)/legal representative(s) of the subject, following written and oral information by the investigator regarding nature, purpose, consequences and possible risks of the clinical study.

Exclusion Criteria

1. History and/or presence of a clinically significant condition/ disorder (e.g. congenital immunodeficiency, surgery, chromosomal abnormalities etc.), which per investigator's judgement may influence the study outcome or the safety of the subject
2. Adverse maternal and/or fetal medical history that may influence:
    a. growth/development of the subject and/or
    b. tolerance of the IP (for the formula groups)
3. Readmission to hospital (except for hyperbilirubinemia) prior to enrolment
4. Self-reported parental and siblings' history of previously medically confirmed and clinically relevant allergy to cow's milk protein
5. Use of intravenously administered antibiotics prior to V1 by the subject.
6. Use of further concurrent treatment (including any natural health products) that may influence the study outcome (e.g. antibiotics, prebiotics, probiotics etc.) during the days prior to V1 and, unless medically indicated and recommended by a medical care provider, during the study as per investigator's judgement; vaccination is allowed
7. Subject participating in another clinical study prior to V1 and during the study

Table S2. Energy, nutrients and HMOs in 5HMO-Mix formula and the control IF, per 100 g formula powder and 100 ml ready-to-drink

	Unit	5HMO-Mix		IF	
		per 100 g powder	per 100 ml ready to drink	per 100 g powder	per 100 ml ready to drink
Energy	KJ	2129	280	2163	283
	kcal	507	67	515	68
Fat	g	27	3.6	27	3.6
Saturated	g	9.3	1.2	9.3	1.2
Monounsaturated	g	12.6	1.7	12.6	1.7
Polyunsaturated	g	5.1	0.7	5.1	0.7
Carbohydrate	g	53	7.2	57	7.2
Lactose	g	39	5.2	39	5.2
Maltodextrin	g	11.65	4.8	16	5.4
5HMO-Mix	g	4.35	0.575	-	-
2′FL	g	2.26	0.299	-	-
3-FL	g	0.57	0.075	-	-
LNT	g	1.13	0.150	-	-
3′-SL	g	0.17	0.023	-	-
6′-SL	g	0.22	0.028	-	-
Protein	g	11	1.4	11	1.4
Salt*	g	0.45	0.06	0.45	0.06
Vitamins					
Vitamin A	μg	476	62.8	476	62.8
Vitamin D	μg	9.0	1.2	9.0	1.2
Vitamin E	mg	12	1.6	12	1.6
Vitamin K	μg	60	7.9	60	7.9
Vitamin C	mg	86	11	86	11
Vitamin B1	mg	0.59	0.077	0.59	0.077
Vitamin B2	mg	1.5	0.20	1.5	0.20
Niacin	mg	3.9	0.51	3.9	0.51
Vitamin B6	mg	0.55	0.073	0.55	0.073
Folic acid	μg	169	22.3	169	22.3
Vitamin B12	μg	1.4	0.18	1.4	0.18
Biotin	μg	22	2.9	22	2.9
Pantothenic acid	mg	5.1	0.67	5.1	0.67
Minerals					
Sodium	mg	180	23.8	180	23.8
Potassium	mg	707	93.4	707	93.4
Chloride	mg	339	44.7	339	44.7
Calcium	mg	409	54.0	409	54.0
Phosphorus	mg	305	40.3	305	40.3
Magnesium	mg	46.5	6.14	46.5	6.14
Iron	mg	4.4	0.58	4.4	0.58
Zinc	mg	3.8	0.50	3.8	0.50
Copper	mg	0.29	0.039	0.29	0.039
Manganese	mg	0.10	0.014	0.10	0.014
Fluoride	mg	<0.070	<0.010	<0.070	<0.010
Selenium	μg	14	1.8	14	1.8
Iodine	μg	103	13.5	103	13.5

Table S3. Complete overview of number of infants in the safety set (n = 311) with adverse events during the study period (V1–V6) categorized by MedDRA v23.1. Primary SOC. Other AEs encompass: investigations, pregnancy, puerperium and perinatal conditions, injury, poisoning and procedural complications, musculoskeletal and connective tissue disorders, eye disorders, neoplasms benign, malignant and unspecified, endocrine disorders, hepatobiliary disorders, immune system disorders, nervous system disorders, psychiatric disorders, reproductive system and breast disorders, vascular disorders, blood and lymphatic system disorders, surgical and medical procedures, renal and urinary disorders

	5HMO-Mix	IF	BM	5HMO-Mix vs IF	5HMO-Mix vs BM	IF vs BM
	n	n	n	p-value	p-value	p-value
Gastrointestinal disorders	64	58	40	0.3975	0.0008	0.0180
Infections and infestations	32	28	34	0.5426	0.8817	0.4486
Congenital, familial and genetic disorders	23	27	9	0.6267	0.0072	0.0016
Skin and subcutaneous tissue disorders	14	21	7	0.2660	0.1129	0.0074
Respiratory, thoracic and mediastinal disorders	10	10	7	1.0000	0.4603	0.6140
General disorders and administration site conditions	6	8	15	0.7831	0.0635	0.1838
Metabolism and nutrition disorders	5	1	3	0.1187	0.4981	0.6214
Investigations	4	1	4	0.4451	1.0000	0.6829
Pregnancy, puerperium and perinatal conditions	4	6	5	0.7478	1.0000	1.0000
Injury, poisoning and procedural complications	3	0	3	0.1214	1.0000	0.2464
Musculoskeletal and connective tissue disorders	3	3	1	1.0000	0.3689	0.6214
Eye disorders	2	3	2	1.0000	1.0000	1.0000
Neoplasms benign, malignant and unspecified	2	1	2	0.6214	1.0000	1.0000
Endocrine disorders	1	0	0	0.4976	0.4976	NA
Hepatobiliary disorders	1	2	3	1.0000	0.6214	1.0000
Immune system disorders	1	2	0	1.0000	0.4976	0.4976
Nervous system disorders	1	1	0	1.0000	0.4976	1.0000
Psychiatric disorders	1	1	1	1.0000	1.0000	1.0000
Reproductive system and breast disorders	1	2	0	1.0000	0.4976	0.4976
Vascular disorders	1	1	0	1.0000	0.4976	1.0000
Blood and lymphatic system disorders	0	1	0	1.0000	NA	1.0000
Surgical and medical procedures	0	1	0	1.0000	NA	1.0000
Renal and urinary disorders	0	0	1	NA	1.0000	1.0000

n=number of subjects,

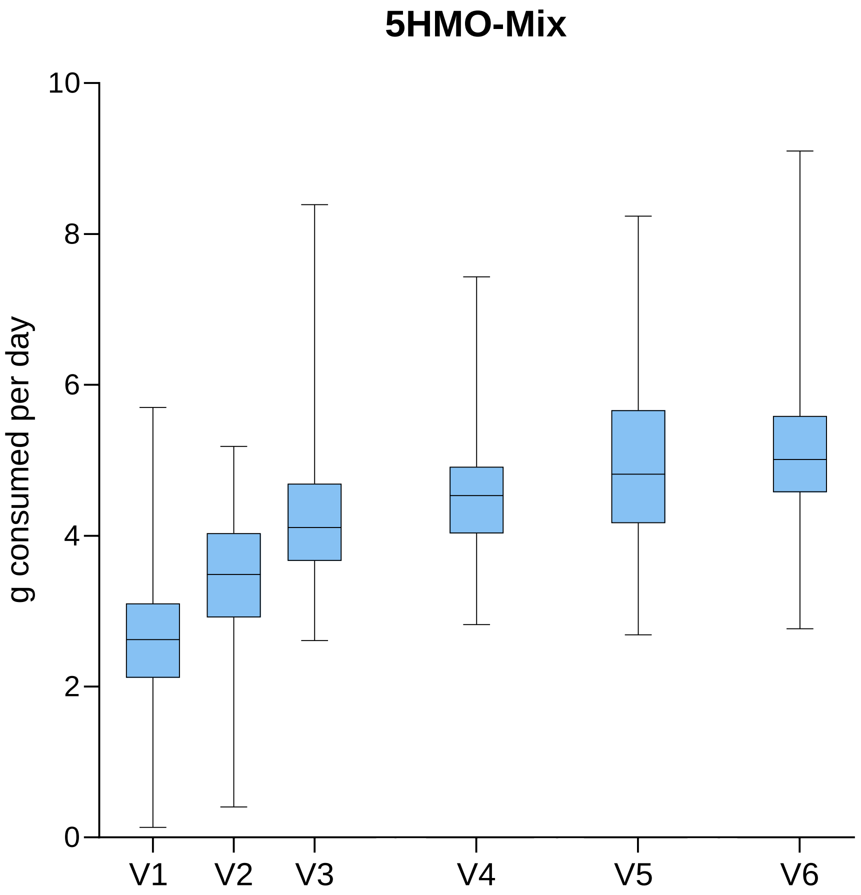

Figure S1. Average intake of the 5HMO-Mix at the different timepoints. Values presented as median, Q1, Q3, min and max. The intake is calculated based on reported quantity (ml) consumed as assessed in the 3-days diary before each visit.

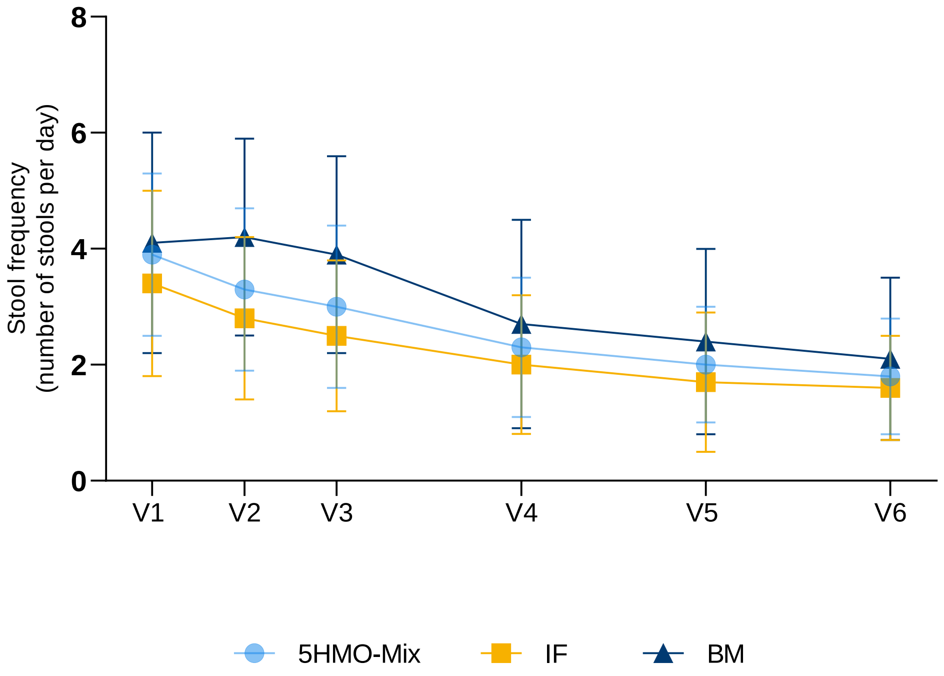

Figure S2. Average stool frequency per day. 5HMO-Mix = Human milk oligosaccharide (HMO) mix in infant formula, IF = infant formula, BM = breast milk. Mean number of stools per day is calculated per subject across the days available for each visit (minimum of one and maximum of three values). Only subjects with at least one stool across the days available for each visit are included in the calculation of descriptive statistics per visit.

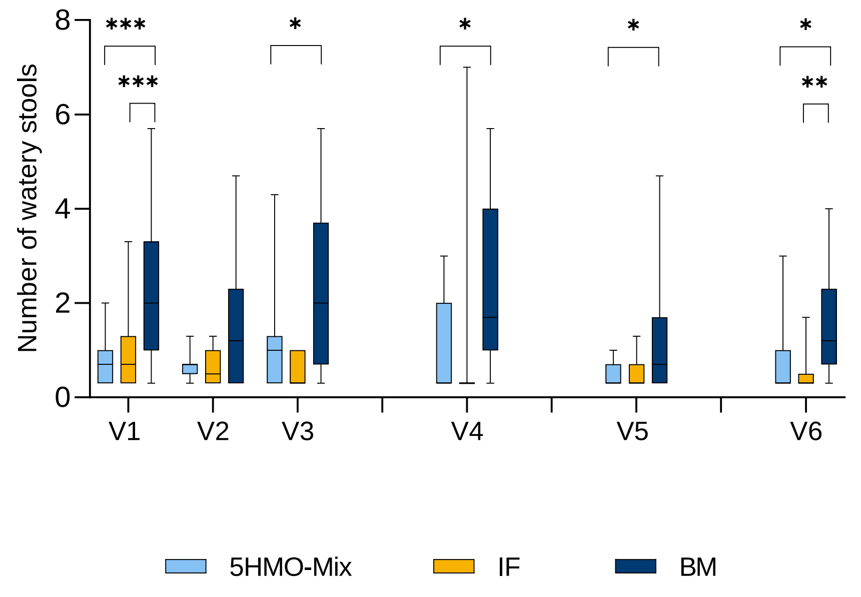
***
***
*
*
*
*
**
Number of watery stools
8
6
4
2
0
V1
V2
V3
V4
V5
V6
5HMO-Mix
IF
BM

(a)

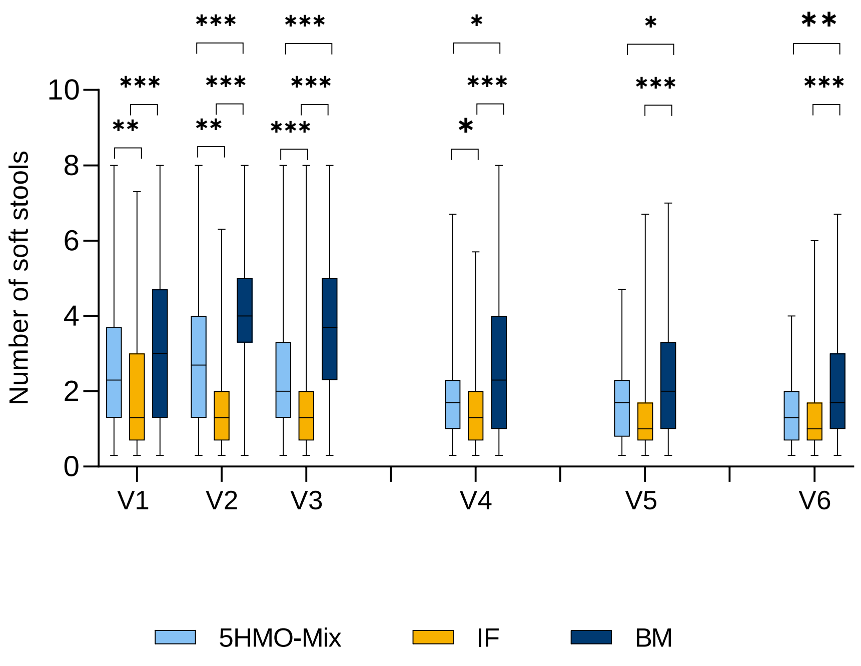
***
***
*
*
**
***
***
***
***
***
***
**
**
***
*
Number of soft stools
10
8
6
4
2
0
V1
V2
V3
V4
V5
V6
5HMO-Mix
IF
BM

(b)

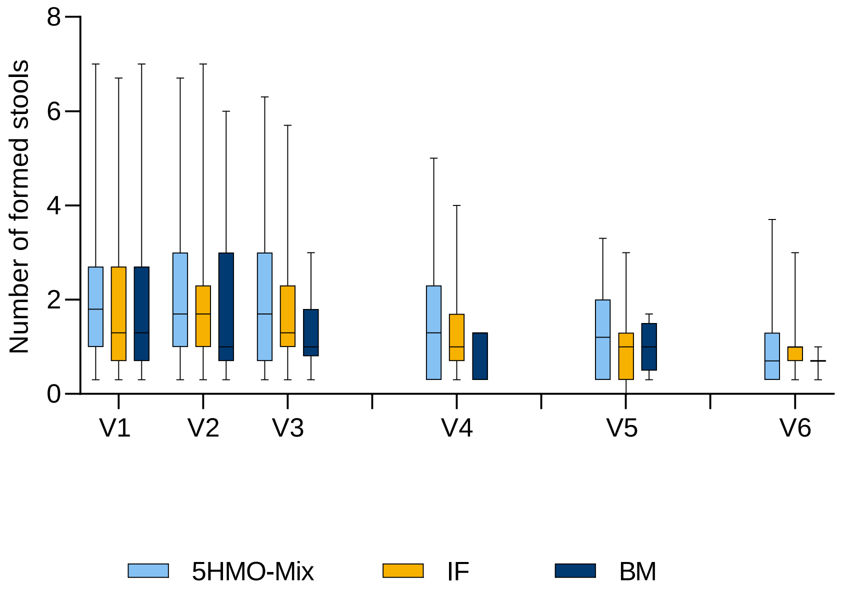

(c)

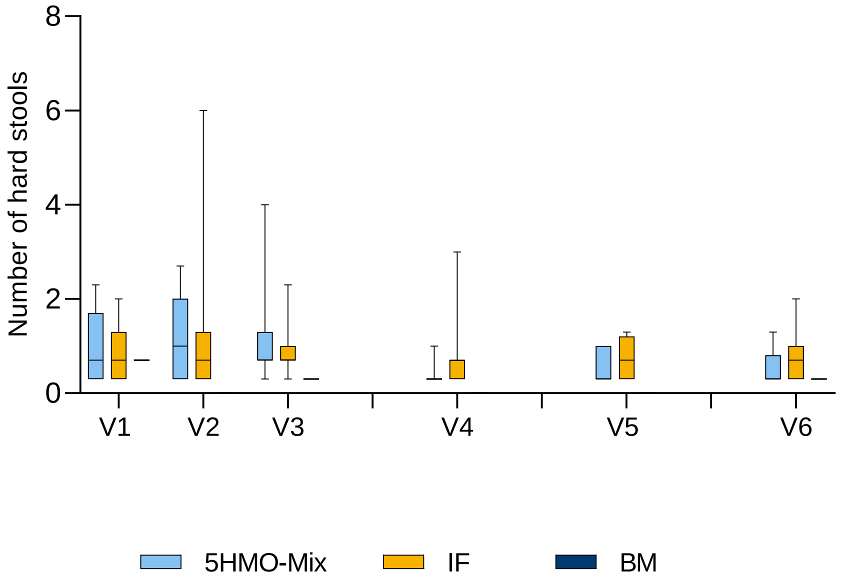

(d)

Figure S3 Stool consistency. Mean total numbers of stools of different consistencies in all three study groups from V1 to V6. (a) Watery stools, (b) Soft stools, (c) Formed stools, and (d) Hard stool. No significant differences observed between groups for formed and hard stools. Mann-Whitney U tests with exploratory p values were calculated to * $p<0.05$, ** $p<0.01$, *** $p<0.001$.

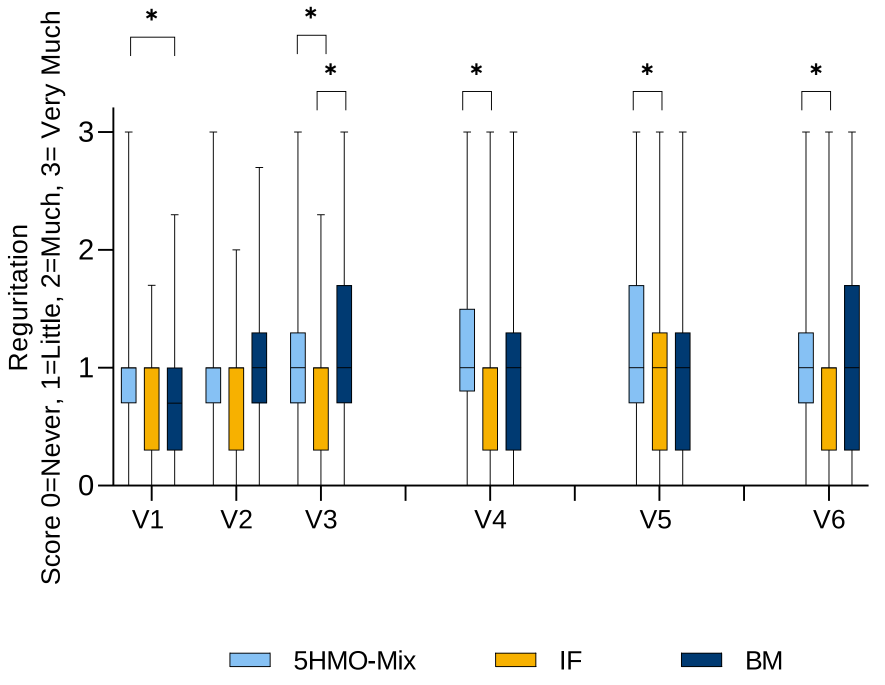
Regurgitation
Score 0=Never, 1=Little, 2=Much, 3= Very Much
0
1
2
3
*
*
*
*
*
*
V1
V2
V3
V4
V5
V6
5HMO-Mix
IF
BM

(a)

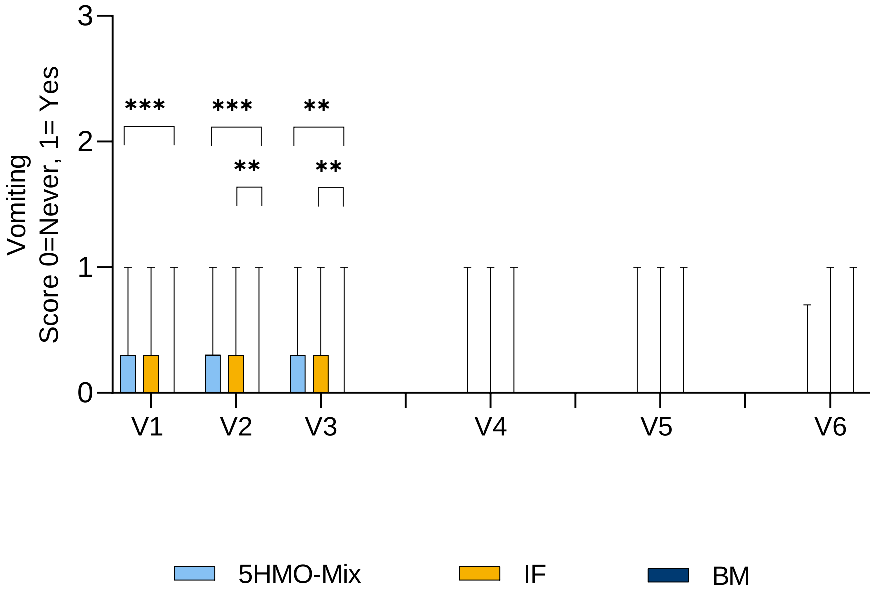
Vomiting
Score 0=Never, 1= Yes
0
1
2
3
***
***
**
**
**
V1
V2
V3
V4
V5
V6
5HMO-Mix
IF
BM

(b)

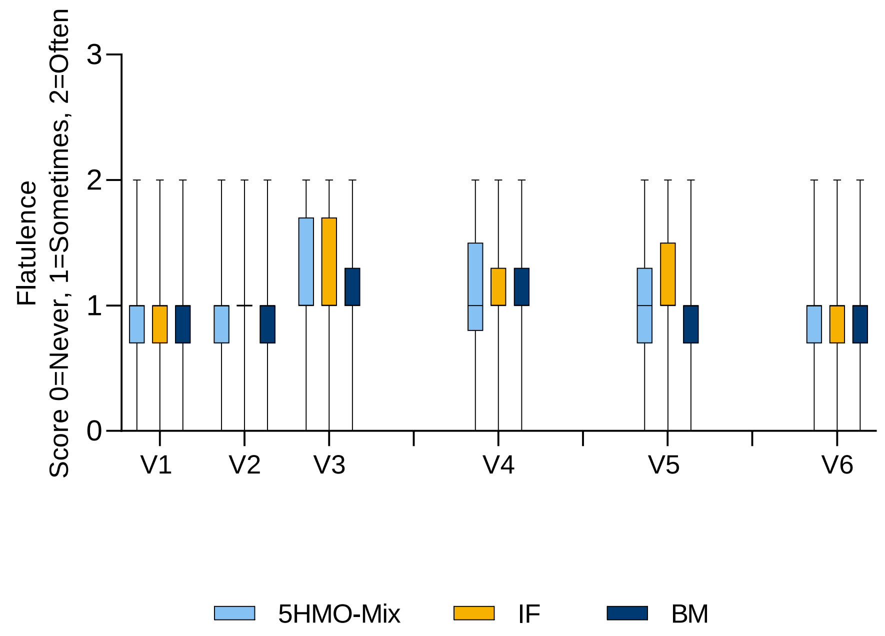

(c)

Figure S4 Score values. Score values for (a) regurgitation (V1–V6). The mean assigned score values for regurgitation are calculated per subject across the days available for each visit (minimum of one and maximum of three values, score values 0=Never, 1=Little, 2=Much, 3= Very Much); (b) Score values for vomiting (V1–V6). Score values 0=Never, 1=Yes; and score values for (c) flatulence (V1–V6), score values 0=Never, 1=Sometimes, 2=Often. Mann-Whitney U tests with exploratory p values were calculated to * $p<0.05$, ** $p<0.01$, *** $p<0.001$.

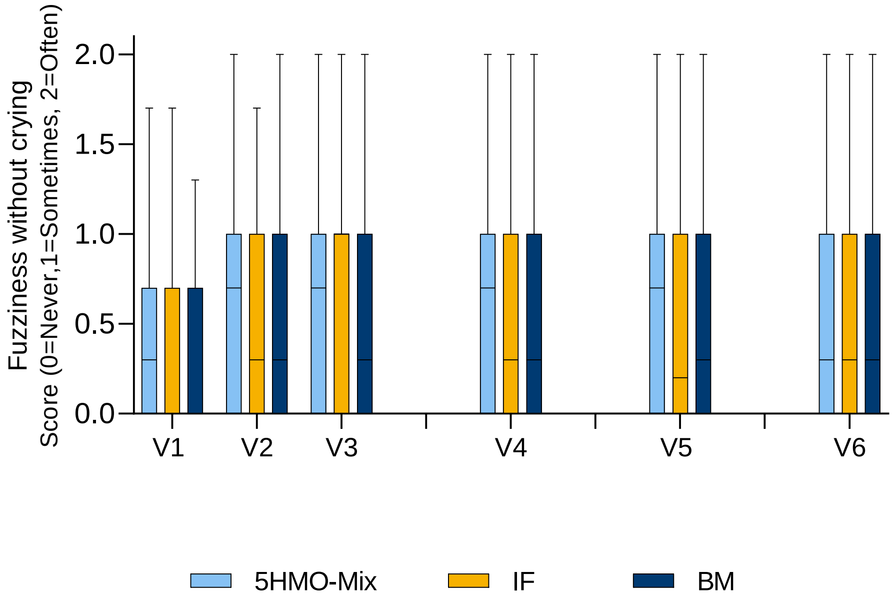
Fuzziness without crying
Score (0=Never,1=Sometimes, 2=Often)
2.0
1.5
1.0
0.5
0.0
V1
V2
V3
V4
V5
V6
5HMO-Mix
IF
BM

(a)

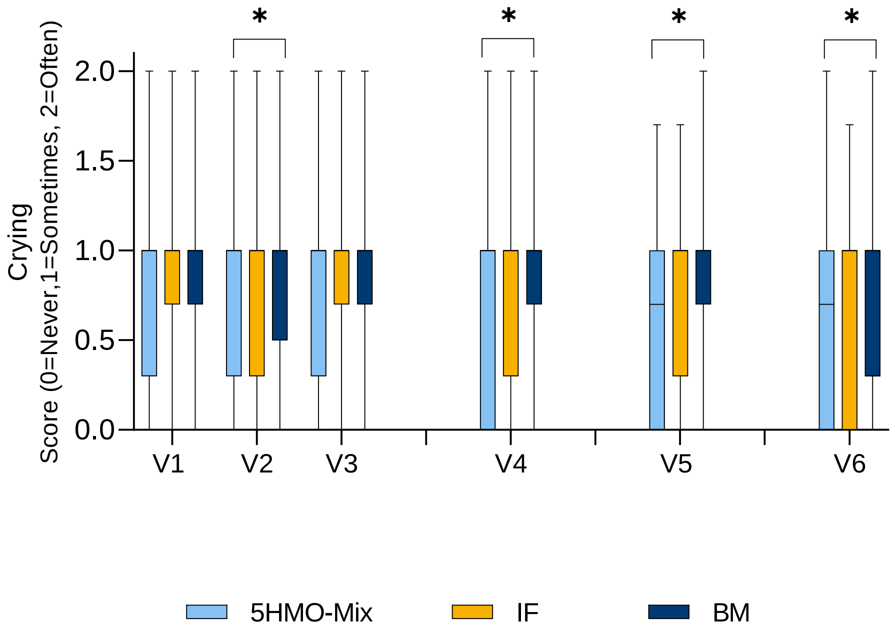
Crying
Score (0=Never,1=Sometimes, 2=Often)
*
*
*
*
2.0
1.5
1.0
0.5
0.0
V1
V2
V3
V4
V5
V6
5HMO-Mix
IF
BM

(b)

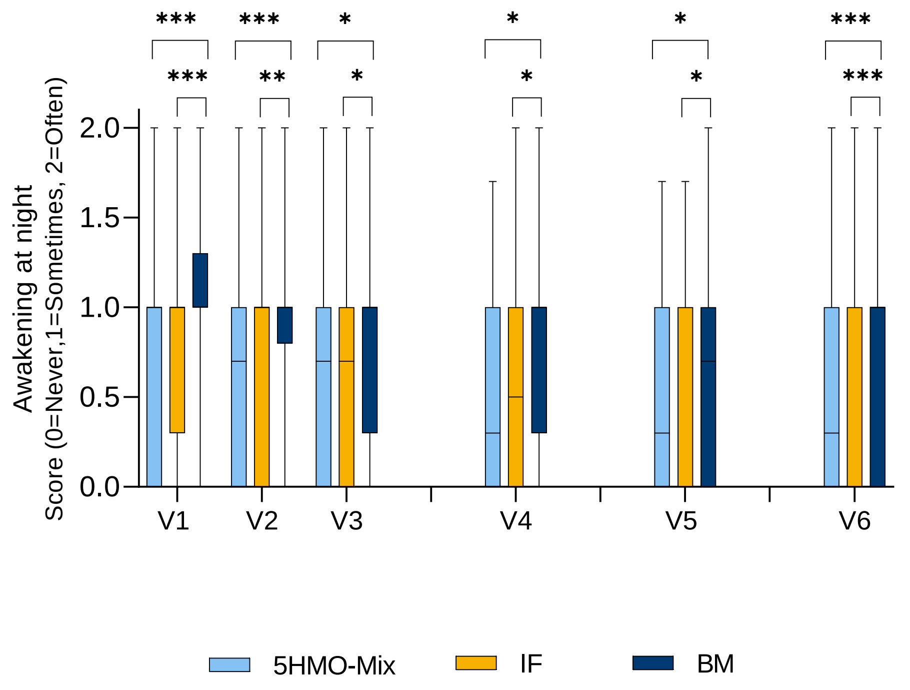

(c)

Figure S5. Score values for behavioral parameters from V1 to V6 from Safety dataset N= 311. (a) Score for fuzziness without crying, (b) Score value for crying, (c) score for awakening at night (V1–V6) in all study groups. Score values 0 =Never, 1=Sometimes and 2=Often. The graphs show the comparison with all three study groups with exploratory p values from Mann-Whitney U tests * p<0.05, ** p<0.01, *** p<0.001.
